# Identification of microRNA signatures in umbilical cord blood associated with maternal characteristics

**DOI:** 10.7717/peerj.6981

**Published:** 2019-05-30

**Authors:** Jaroslav Juracek, Pavel Piler, Petr Janku, Lenka Radova, Ondrej Slaby

**Affiliations:** 1 Central European Institute of Technology (CEITEC), Masaryk University, Brno, Czech Republic; 2 Research Centre for Toxic Compounds in the Environment (RECETOX), Faculty of Science, Masaryk University, Brno, Czech Republic; 3 Department of Gynecology and Obstetrics, Institutions shared with the Faculty Hospital Brno, Institutions of Reproductive Medicine, Faculty of Medicine, Masaryk University, Brno, Czech Republic

**Keywords:** Umbilical cord blood, microRNAs, Maternal characteristics

## Abstract

**Background:**

Umbilical cord blood could serve as useful source of blood markers enabling more efficient and reliable prenatal and neonatal diagnostics. MicroRNAs (miRNAs) are ubiquitous in body fluids where they were used for detecting and monitoring various physiological and pathological conditions. In this descriptive study, we aimed to identify changes in miRNA expression profiles associated with basic maternal somatic and epidemiological characteristics.

**Methods:**

Study is based on 24 mothers from the Pilot phase of CELSPAC: TNG (Central European Longitudinal Studies of Parents and Children: The Next Generation) study. Cord blood was collected at time of delivery and global miRNA profiling was performed using microRNA Ready-to-use PCR Human Panel I+II TaqMan microarrays. Expression profiles were statistically evaluated in relation to maternal age, BMI, pregnancy weight gain, blood type, Rh factor status, allergies during pregnancy, addictive substance abuse and smoking status.

**Results:**

We analyzed expression of 752 human mature miRNAs in 24 samples of umbilical cord blood. For all maternal characteristics tested we described a specific signature of significantly deregulated miRNAs (*P* < 0.05). Analysis revealed seven miRNA associated with maternal age (three increased and four decreased in women younger than 35 years), 14 miRNAs associated with BMI status (five miRNAs increased and nine miRNAs decreased in women with BMI > 25) and nine miRNAs associated with maternal weight gain during pregnancy (eight miRNAs increased, and one miRNA decreased in women with weight gain < 12 kg). Additionally, 17 miRNAs correlated to blood type (two miRNAs decreased in blood type A, 11 increased in blood type B, two miRNAs increased in blood type AB and two miRNAs increased in blood type 0) and 17 miRNAs to Rh status of mother. We also detected seven miRNAs deregulated in umbilical cord blood of women with allergy (four increased and three decreased in women with allergy), four miRNAs associated to addictive substance abuse status (two up- and two downregulated in women with addictive substance abuse) and eight miRNAs associated with maternal cigarette smoking during pregnancy.

**Conclusions:**

We successfully described differences in miRNA profiles in umbilical cord blood associated with basic characteristics connected with mother. Our data suggest that miRNAs in umbilical cord blood are detectable and associated with a wide range of maternal characteristics. These results indicate that miRNAs could potentially serve, and should be studied, as biomarkers for screening and diagnosis of pregnancy-associated complications and pathologies.

## Introduction

Umbilical cord blood (UCB) is blood that remains in the placenta and umbilical cord after birth (Waller-Wise, 2011). Apart from common blood elements cord blood is a rich source of primitive, undifferentiated hematopoietic stem cells (Erices, Conget & Minguell, 2000). Though it was originally considered as a waste product, it has developed into an important allogeneic donor source in transplantation in pediatrics and a novel source of blood markers (O’Brien, Tiedemann & Vowels, 2006). Especially in neonatal diagnostics, where blood from peripheral veins is used, UCB might be a suitable alternative and valuable source of blood biomarkers thanks to noninvasive and painless collection. Recent studies show that certain acute phase reactants are elevated in umbilical cord blood of premature infants with early onset sepsis (Mithal et al., 2017). Other studies described the distribution of immune biomarkers in cord blood across gestational age and show the association between biomarker level patterns and preterm birth (Matoba et al., 2009). Similarly, growth factors levels in cord blood can correlate with birth weight and postnatal growth in premature infants and was also associated with risk for postnatal growth failure (Voller et al., 2014).

One of the most abundant groups of biomarkers are microRNAs (miRNAs). They are ubiquitous in most of the body fluid types, where they may have functional roles associated with the surrounding tissues (Weber et al., 2010). In addition, the changes in levels of specific miRNAs in body fluids were used for detecting and monitoring various somatic and pathological conditions (Cortez et al., 2011; Velu, Ramesh & Srinivasan, 2012). The role of circulating miRNAs has been reported also in the context of neonatal diagnostics. Higher expression levels of miR-615-3p were observed in neonatal peripheral blood where this miRNA promoted acute respiratory distress syndrome (ARDS) development (Wu & Ding, 2018). Similarly, a decrease in levels of miR-132 and miR-223 was associated with neonatal sepsis (Dhas, Dirisala & Bhat, 2018).

The first description of miRNA profiling in cord blood was reported in 2015. In this study, downregulation of miR-374a-5p was observed in infants with hypoxic ischemic encephalopathy (HIE) (Looney et al., 2015). In a subsequent study, altered miRNA levels were detected also in umbilical cord blood of neonates with perinatal asphyxia (PA), suggesting their potential role in early detection of this disease (O’Sullivan et al., 2018). Moreover, previous research has shown that not only pathologies but also somatic characteristic such as birth weight modifies the expression of miRNAs (Rodil-Garcia et al., 2017). Based on these observations, we hypothesize that specific miRNA patterns in umbilical cord blood can be associated with physiological as well as pathological conditions of mother or fetus.

In this pilot study, we aimed to perform high-capacity screening of miRNA levels in umbilical cord blood in order to describe miRNA signatures associated with basic maternal somatic and epidemiological characteristics.

## Materials & Methods

### Patient cohorts

Study data and samples were obtained from the Pilot phase of CELSPAC: TNG (Central European Longitudinal Studies of Parents and Children: The Next Generation Study). CELSPAC: TNG is designed as a new prospective birth cohort which will follow up on 10,000 children from their prenatal period to adolescence with the aim of assessing multiple factors potentially affecting children’s health. The Ethical committee of University Hospital Brno, Czech Republic approved this study (No. 20140409-01). All mothers gave their written informed consent.

The Pilot phase of CELSPAC: TNG was initiated in April 2015 to evaluate feasibility of the protocol for collection, processing and storing of biological samples (cord blood; venous blood, urine and buccal smear from mothers; stool, dry blood spot and buccal smear from babies); to estimate future study response rates; to evaluate on-line distribution and respond rate of questionnaires.

The current study included 24 mothers from whom we collected umbilical cord blood (UCB) at time of delivery. We also used the data from medical records related to mother somatic characteristics, pregnancy and birth. Women with non-physiological pregnancy including medical and obstetrical complications or comorbid conditions that could affect fetus were excluded.

### Sample collection and processing

Cord blood was collected to S-Monovette^®^ K3E (S-Monovette^®^ 9 ml, K3 EDTA) after the second stage of labor from the umbilical cord vein. Plasma was prepared by centrifugation (2,500 g for 10 min at 22 °C) and aliquoted into tubes as 250 µL samples and stored at −80 °C.

### miRNA Quantification

Prior to RNA isolation umbilical cord blood plasma samples were centrifuged at 4 °C at 1,000 g for 5 min. Total RNA from 200 µl of UCB plasma was isolated using miRNeasy Serum/Plasma Kit (Qiagen, Hilden, Germany). RNA quality and quantity was evaluated using Nanodrop 2000 Spectrophotometer (Thermo Fisher Scientific, Waltham, MA, USA). Whole-genome miRNA profiling was performed by use of Human panel I+II, V4, miRCURY LNA miRNA miRNome PCR Panel (Exiqon by Qiagen, Hilden, Germany) accordingly to the manufacturers protocol. On each plate for each sample we included interplate calibrator enabling compensation of signal variations between instrument runs (inter-plate calibrator assay UniSp3). Expression levels of miRNA represented by Ct values were normalized on U6 reference gene expression level using the 2^−ΔCt^ method where ΔCt = (Ct_Target miRNA − Ct_U6). Relative miRNA expression levels were correlated with selected epidemiological and somatic characteristics of mothers. Only miRNAs having non-zero expression values within more than 50% of samples were included in statistical analysis. Statistical analyses were performed within R/Bioconductor environment. The Mann–Whitney and Kruskal–Wallis test were applied for two or more categorial variables. In all comparisons, *p*-values <0.05 were set as statistically significant.

## Results

Global profiling performed using TaqMan array enabling detection of 752 human mature miRNAs was performed in 24 umbilical cord blood plasma samples (Dataset S1). From this number, 656 miRNAs were detectable in at least one sample and 491 miRNAs had non-zero expression values within more than 50% of samples. For subsequent statistical analysis, samples were regrouped by selected maternal characteristics including age, BMI, pregnancy weight gain (PWG), blood type, Rh factor status, allergies during pregnancy, addictive substance abuse and smoking status (summarized in Table 1). Genome-wide microRNA expression data have been deposited in the Gene Expression Omnibus repository under accession number GSE128943.

**Table 1:** List of maternal characteristics used in specific statistical analysis.

Maternal characteristics	Specific characteristics (Number of subjects)					
Age	<35 years (15)			>35 years (9)		
BMI	<25 (19)			>25 (5)		
Pregnancy weight gain	<12 kg (12)			>12 kg (12)		
Blood type	A (10)	B (4)		0 (2)		AB (5)
Rh factor	Negative (6)			Positive (18)		
Allergies	Yes (12)			No (12)		
Addictive substance abuse	Yes (9)			No (15)		
Smoking status	Non-smoker (15)		Smoker (3)		Stop-smoker (6)	

Global expression analysis revealed pattern of seven miRNA associated with maternal age namely miR-137, miR-665, miR-770-5p were increased and miR-625-3p, miR-377-3p, miR-224-3p and miR-671-3p decreased in women younger than 35 years. Next, we analyzed miRNA changes in relation to a maternal BMI. Analysis identified 14 miRNAs with differential levels between women with overweight (BMI > 25) and women with normal BMI status (BMI 18.5–25). Five of these miRNAs were increased and nine miRNAs decreased in women with overweight (BMI > 25). Similarly, levels of nine miRNAs were amended in umbilical cord blood in association with maternal weight gain during pregnancy (eight miRNAs increased, and one miRNA decreased in women with pregnancy weight gain lesser than 12 kg). Additionally, we identified 17 miRNAs with levels correlated to blood type of mother (two miRNAs showed decreased levels in blood type A group, 11 showed increased levels in blood type B group, two miRNAs were increased in blood type AB group and two miRNAs were increased in blood type 0 group). Moreover, 17 miRNAs were significantly increased when samples of Rh positive and Rh-negative women were compared. In mothers with unspecified allergies we detected pattern of seven miRNAs with deregulated levels; miR-181d-5p, miR-545-3p, miR-153-3p, miR-632 increased in women with allergy, miR-371a-3p, miR-96-5p, miR-216a-5p decreased in women with allergy. Next, we described four miRNAs with levels associated to addictive substance abuse status. Subsequent multivariate statistical analysis revealed increased levels of six miRNAs (miR-129-5p, miR-30b-3p, miR-187-3p, miR-507, miR-520b and miR-33b-3p) associated with maternal cigarette smoking during pregnancy. Similarly, we identified significantly higher level of miR-138-1-3p and decreased level of miR-760 in mothers who quit smoking during pregnancy. Complete list of deregulated miRNAs associated with monitored maternal characteristics is summarized in Table 2 (detailed information is Tables S1–S8).

**Table 2:** List of umbilical cord blood miRNAs significantly associated with individual maternal characteristics (*P* < 0.05).

Maternal characteristics	List of associated miRNAs
Age*(<35 years)*	↑ miR-137, miR-665, miR-770-5p; ↓ miR-625-3p, miR-377-3p, miR-224-3p, miR-671-3p.
BMI*(>25)*	↑ miR-1203, miR-143-3p, miR-582-5p, miR-510-5p, miR-450a-5p; ↓ miR-604, miR-205-5p, miR-551a, miR-203a, miR-548l, miR-424-5p, miR-627-5p, miR-629-3p, miR-141-3p.
Pregnancy weight gain*(<12 kg)*	↑ miR-138-5p, miR-760, miR-9-3p, miR-548c-5p, miR-1260a, miR-145-3p, miR-34a-3p, miR-320d; ↓ miR-1224-3p.
Blood type	A: ↓ miR-380-5p, miR-92a-1-5p;B: ↑miR-760, miR-10b-5p, miR-34b-3p, miR-145-5p, miR-153-3p, miR-548c-5p, miR-511-5p, miR-330-5p, miR-24-1-5p, let-7b-3p, let-7f-2-3p; AB: ↑ miR-595, miR-431-3p; 0: ↑ miR-641, miR-548h-5p.
Rh factor status*(positive)*	↑ miR-141-3p, miR-188-5p, miR-211-5p, miR-205-5p, miR-150-5p, miR-181c-5p, miR-124-3p, miR-142-5p, miR-15b-5p, miR-1269a, miR-1260a, miR-518d-3p, miR-27a-5p; ↓ miR-514a-3p, miR-449b-5p, miR-641, miR-548l.
Allergies*(Yes)*	↑ miR-181d-5p, miR-545-3p, miR-153-3p, miR-632; ↓ miR-371a-3p, miR-96-5p, miR-216a-5p.
Addictive substance abuse*(Yes)*	↑ miR-138-1-3p, miR-33b-3p; ↓ miR-760, miR-377-3p.
Smoking status*(Yes)*	↑ miR-129-5p, miR-30b-3p, miR-187-3p, miR-507, miR-520b, miR-33b-3p, miR-138-1-3p; ↓ miR-760.

## Discussion

Circulating miRNAs are currently widely accepted as promising markers of both pathological and physiological conditions (Velu, Ramesh & Srinivasan, 2012; Kosaka, Iguchi & Ochiya, 2010; Mitchell et al., 2008). In reproductive medicine specific miRNA profiles in peripheral blood were found to relate to complications of pregnancy, such as placental abruption (Miura et al., 2017), ectopic pregnancy (Zhao et al., 2012) or preeclampsia (Gunel et al., 2011). Moreover, expression patterns of circulating miRNA are promising solution for noninvasive prenatal testing of Down Syndrome and other genetic diseases (Erturk et al., 2016). However, considering direct connection with fetus, umbilical cord blood could serve as valuable source of biomarkers in prenatal diagnostics and screening.

In our pilot study, we successfully described aberration in miRNA profiles in umbilical cord blood plasma associated with basic characteristics connected with mother. So far publications focused on the identification of miRNA expression profiles in umbilical cord blood were connected mainly to pathophysiology of a particular disease or pathological conditions. As in the case of infants with hypoxic ischemic encephalopathy (HIE) where miR-374a revealed significant down-regulation in cord blood of infants with perinatal asphyxia and subsequent HIE (Looney et al., 2015). Similarly study of Rager et al. highlight miRNAs as novel responders to prenatal arsenic exposure that may contribute to associated immune response perturbations (Rager et al., 2014). Despite diagnostic potential of UBC there is lack of descriptive studies which demonstrate miRNA deregulation in association with basic somatic and epidemiological characteristics of mothers and newborns. Ghaffari et al. investigated whether maternal obesity is associated with alterations in expression of fetal miRNAs (Ghaffari et al., 2015). Despite negative results, this study delineated role of miRNA within delivery course and success rate.

Since umbilical cord blood flow is dynamic and progressive process where exchange of blood elements and nutrients occurs, we expected that possible deregulation should be influenced by both the mother’s and the newborn’s environment. Currently there are no comparable studies supporting our findings, however, we found overlap in identified miRNAs within studies focusing on miRNAs functioning. For example, miR-625-3p and miR-671-3p showing significant association with maternal age in our study were described also in study of Huan et al. as age-associated miRNAs (Huan et al., 2018). Moreover, miR-671-3p seems to be differentially expressed between keratinocytes prepared from child skin and aged skin (Muther et al., 2017). Similarly, we identified miRNAs indicating association with maternal BMI or weight gain during pregnancy. In accordance with our findings, miR-143-3p was depicted as regulator of adipocyte differentiation (Esau et al., 2004) and its upregulation in mesenteric fat in mice was associated with body weight (Takanabe et al., 2008). MiR-450-5p, miR-203a, miR-141-3p and miR-205-5p were found to be differentially expressed in subcutaneous adipose tissue of obese individuals and normal-weight subjects (Kurylowicz et al., 2018). Other miRNAs such as miR-551a or miR-138-5p were already associated with BMI (Iacomino et al., 2019) or weight gain (Zhao et al., 2017). Regarding to mothers with allergies, we identified miR-181d-5p, member of miR-181 family, which has a central role in vascular inflammation by controlling critical signaling pathways and regulates immune cell homeostasis (Sun, Sit & Feinberg, 2014). Other identified molecule, miR-371a-3p was suggested to modulate the Th1/Th2 balance in asthma (Qiu et al., 2017). Among miRNAs deregulated within mothers who did smoke during pregnancy we described miR-129-5p which was observed to be upregulated in lung cancer patients with a smoking history (Momi et al., 2014; Vucic et al., 2014). Further, miR-33b-3p was differentially expressed between rectal cancer tissue and normal rectal mucosa and associated with smoking and miR-520b was significantly differentially expressed with cigarette smoking and associated with CIMP and/or MSI status in colon and rectal cancer (Mullany et al., 2016).

## Conclusion

The data obtained in this pilot study indicated that miRNA levels in umbilical cord blood plasma are related to somatic and epidemiological characteristics of mother and newborn infants. Therefore, UCB miRNAs should be studied also as biomarkers for screening and diagnosis of pregnancy-associated complications and pathologies.

##  Supplemental Information

10.7717/peerj.6981/supp-1 Supplemental Information 1 Supplementary Tables S1–S8 Detailed information of individual maternal characteristics and miRNA differences presented as median difference and *p*-value.

10.7717/peerj.6981/supp-2 Table S9 Samples stratification into various clinico-epidemiological categories

10.7717/peerj.6981/supp-3 Dataset S1 Raw microRNA expression data

## Additional Information and Declarations

### Data Availability

The following information was supplied regarding data availability: The raw measurements are available in Dataset S1 and Table S9. In Table S9, all samples are stratified into the clinico-epidemiological categories tested in the study (e.g., age, pregnancy weight gain, blood type, Rh factor, etc.). In Dataset S1, the global expression profiles of microRNAs are presented as Ct values for each sample.

## References

[ref-1] Cortez MA, Bueso-Ramos C, Ferdin J, Lopez-Berestein G, Sood AK, Calin GA (2011). MicroRNAs in body fluids–the mix of hormones and biomarkers. Nature Reviews. Clinical Oncology.

[ref-2] Dhas BB, Dirisala VR, Bhat BV (2018). Expression levels of candidate circulating microRNAs in early-onset neonatal sepsis compared with healthy newborns. Genomics Insights.

[ref-3] Erices A, Conget P, Minguell JJ (2000). Mesenchymal progenitor cells in human umbilical cord blood. British Journal of Haematology.

[ref-4] Erturk B, Karaca E, Aykut A, Durmaz B, Guler A, Buke B, Yeniel AO, Ergenoglu AM, Ozkinay F, Ozeren M, Kazandi M, Akercan F, Sagol S, Gunduz C, Cogulu O (2016). Prenatal evaluation of MicroRNA expressions in pregnancies with down syndrome. BioMed Research International.

[ref-5] Esau C, Kang X, Peralta E, Hanson E, Marcusson EG, Ravichandran LV, Sun Y, Koo S, Perera RJ, Jain R, Dean NM, Freier SM, Bennett CF, Lollo B, Griffey R (2004). MicroRNA-143 regulates adipocyte differentiation. The Journal of Biological Chemistry.

[ref-6] Ghaffari N, Parry S, Elovitz MA, Durnwald CP (2015). The effect of an obesogenic maternal environment on expression of fetal umbilical cord blood miRNA. Reproductive Sciences.

[ref-7] Gunel T, Zeybek YG, Akcakaya P, Kalelioglu I, Benian A, Ermis H, Aydinli K (2011). Serum microRNA expression in pregnancies with preeclampsia. Genetics and Molecular Research.

[ref-8] Huan T, Chen G, Liu C, Bhattacharya A, Rong J, Chen BH, Seshadri S, Tanriverdi K, Freedman JE, Larson MG, Murabito JM, Levy D (2018). Age-associated microRNA expression in human peripheral blood is associated with all-cause mortality and age-related traits. Aging Cell.

[ref-9] Iacomino G, Russo P, Marena P, Lauria F, Venezia A, Ahrens W, De Henauw S, De Luca P, Foraita R, Gunther K, Lissner L, Molnar D, Moreno LA, Tornaritis M, Veidebaum T, Siani A (2019). Circulating microRNAs are associated with early childhood obesity: results of the I.Family Study. Genes & Nutrition.

[ref-10] Kosaka N, Iguchi H, Ochiya T (2010). Circulating microRNA in body fluid: a new potential biomarker for cancer diagnosis and prognosis. Cancer Science.

[ref-11] Kurylowicz A, Wicik Z, Owczarz M, Jonas MI, Kotlarek M, Swierniak M, Lisik W, Jonas M, Noszczyk B, Puzianowska-Kuznicka M (2018). NGS reveals molecular pathways affected by obesity and weight loss-related changes in mirna levels in adipose tissue. International Journal of Molecular Sciences.

[ref-12] Looney AM, Walsh BH, Moloney G, Grenham S, Fagan A, O’Keeffe GW, Clarke G, Cryan JF, Dinan TG, Boylan GB, Murray DM (2015). Downregulation of umbilical cord blood levels of miR-374a in neonatal hypoxic ischemic encephalopathy. The Journal of Pediatrics.

[ref-13] Matoba N, Yu Y, Mestan K, Pearson C, Ortiz K, Porta N, Thorsen P, Skogstrand K, Hougaard DM, Zuckerman B, Wang X (2009). Differential patterns of 27 cord blood immune biomarkers across gestational age. Pediatrics.

[ref-14] Mitchell PS, Parkin RK, Kroh EM, Fritz BR, Wyman SK, Pogosova-Agadjanyan EL, Peterson A, Noteboom J, O’Briant KC, Allen A, Lin DW, Urban N, Drescher CW, Knudsen BS, Stirewalt DL, Gentleman R, Vessella RL, Nelson PS, Martin DB, Tewari M (2008). Circulating microRNAs as stable blood-based markers for cancer detection. Proceedings of the National Academy of Sciences of the United States of America.

[ref-15] Mithal LB, Palac HL, Yogev R, Ernst LM, Mestan KK (2017). Cord blood acute phase reactants predict early onset neonatal sepsis in preterm infants. PLOS ONE.

[ref-16] Miura K, Higashijima A, Murakami Y, Fuchi N, Tsukamoto O, Abe S, Hasegawa Y, Miura S, Masuzaki H (2017). Circulating levels of pregnancy-associated, placenta-specific micrornas in pregnant women with placental abruption. Reproductive Sciences.

[ref-17] Momi N, Kaur S, Rachagani S, Ganti AK, Batra SK (2014). Smoking and microRNA dysregulation: a cancerous combination. Trends in Molecular Medicine.

[ref-18] Mullany LE, Herrick JS, Wolff RK, Stevens JR, Slattery ML (2016). Association of cigarette smoking and microRNA expression in rectal cancer: insight into tumor phenotype. Cancer Epidemiology.

[ref-19] Muther C, Jobeili L, Garion M, Heraud S, Thepot A, Damour O, Lamartine J (2017). An expression screen for aged-dependent microRNAs identifies miR-30a as a key regulator of aging features in human epidermis. Aging.

[ref-20] O’Brien TA, Tiedemann K, Vowels MR (2006). No longer a biological waste product: umbilical cord blood. The Medical Journal of Australia.

[ref-21] O’Sullivan MP, Looney AM, Moloney GM, Finder M, Hallberg B, Clarke G, Boylan GB, Murray DM (2018). validation of altered umbilical cord blood microrna expression in neonatal hypoxic-ischemic encephalopathy. JAMA Neurology.

[ref-22] Qiu YY, Zhang YW, Qian XF, Bian T (2017). miR-371, miR-138, miR-544, miR-145, and miR-214 could modulate Th1/Th2 balance in asthma through the combinatorial regulation of Runx3. American Journal of Translational Research.

[ref-23] Rager JE, Bailey KA, Smeester L, Miller SK, Parker JS, Laine JE, Drobna Z, Currier J, Douillet C, Olshan AF, Rubio-Andrade M, Styblo M, Garcia-Vargas G, Fry RC (2014). Prenatal arsenic exposure and the epigenome: altered microRNAs associated with innate and adaptive immune signaling in newborn cord blood. Environmental and Molecular Mutagenesis.

[ref-24] Rodil-Garcia P, Arellanes-Licea EDC, Montoya-Contreras A, Salazar-Olivo LA (2017). Analysis of MicroRNA expression in newborns with differential birth weight using newborn screening cards. International Journal of Molecular Sciences.

[ref-25] Sun X, Sit A, Feinberg MW (2014). Role of miR-181 family in regulating vascular inflammation and immunity. Trends in Cardiovascular Medicine.

[ref-26] Takanabe R, Ono K, Abe Y, Takaya T, Horie T, Wada H, Kita T, Satoh N, Shimatsu A, Hasegawa K (2008). Up-regulated expression of microRNA-143 in association with obesity in adipose tissue of mice fed high-fat diet. Biochemical and Biophysical Research Communications.

[ref-27] Velu VK, Ramesh R, Srinivasan AR (2012). Circulating MicroRNAs as Biomarkers in Health and Disease. Journal of Clinical and Diagnostic Research.

[ref-28] Voller SB, Chock S, Ernst LM, Su E, Liu X, Farrow KN, Mestan KK (2014). Cord blood biomarkers of vascular endothelial growth (VEGF and sFlt-1) and postnatal growth: a preterm birth cohort study. Early Human Development.

[ref-29] Vucic EA, Thu KL, Pikor LA, Enfield KS, Yee J, English JC, MacAulay CE, Lam S, Jurisica I, Lam WL (2014). Smoking status impacts microRNA mediated prognosis and lung adenocarcinoma biology. BMC Cancer.

[ref-30] Waller-Wise R (2011). Umbilical cord blood: information for childbirth educators. The Journal of Perinatal Education.

[ref-31] Weber JA, Baxter DH, Zhang S, Huang DY, Huang KH, Lee MJ, Galas DJ, Wang K (2010). The microRNA spectrum in 12 body fluids. Clinical Chemistry.

[ref-32] Wu YQ, Ding YJ (2018). Overexpressed microRNA-615-3p promotes progression of neonatal acute respiratory distress syndrome by inhibiting differentiation of mesenchymal stem cells to alveolar type II epithelial cells. European Review for Medical and Pharmacological Sciences.

[ref-33] Zhao H, Shen J, Daniel-MacDougall C, Wu X, Chow WH (2017). Plasma MicroRNA signature predicting weight gain among Mexican-American women. Obesity.

[ref-34] Zhao Z, Zhao Q, Warrick J, Lockwood CM, Woodworth A, Moley KH, Gronowski AM (2012). Circulating microRNA miR-323-3p as a biomarker of ectopic pregnancy. Clinical Chemistry.

