# Supplementary material for: Identification of microRNA signatures in umbilical cord blood associated with maternal characteristics
Source: PeerJ. 2019 May 30;7:e6981. doi: 10.7717/peerj.6981 (PMC6545228; doi:10.7717/peerj.6981)
Supplement: Supplemental Information 1 — Detailed information of individual maternal characteristics and miRNA differences presented as median difference and p-value. [file peerj-07-6981-s001.docx]

**Supplementary information**

Detailed information of individual maternal characteristics and miRNA differences presented as median difference and p-value are provided in Supplementary Tables S1-S8.

Supplementary Table S1 MicroRNAs with significantly different levels in umbilical cord blood of mothers older than 35 years.

| MiRNA | FC | 95% Confidence of FC | Median difference | p-value |
| --- | --- | --- | --- | --- |
| miR−137 | -0.027 | ( -0.053 ; -0.002) | -0.009 | 0.0198 |
| miR−625−3p | -0.374 | (-2.580 ; 1.832) | -0.632 | 0.0408 |
| miR−665 | -0.070 | (-0.145 ; 0.005) | -0.008 | 0.0397 |
| miR−377−3p | 0.037 | (-0.111 ; 0.184) | 0.082 | 0.0475 |
| miR−770−5p | -0.077 | (-0.140 ; -0.015) | -0.066 | 0.0468 |
| miR−224−3p | -0.165 | (-2.471 ; 2.140) | -0.486 | 0.0408 |
| miR−671−3p | 0.0417 | (-0.209 ; 0.292) | 0.131 | 0.0144 |

Supplementary Table S2 MicroRNAs with significantly different levels in umbilical cord blood of mothers with overweight (BMI>25).

| MiRNA | FC | 95% Confidence of FC | Median difference | p-value |
| --- | --- | --- | --- | --- |
| miR-1203 | 0.006 | (-0.011 ; 0.024) | 0.004 | 0.0031 |
| miR-143-3p | -16.705 | (-30.303 ; -3.107) | -8.776 | 0.0039 |
| miR-582-5p | -0.551 | (-0.941 ; -0.160) | -0.440 | 0.0049 |
| miR-510-5p | 0.013 | (-0.022 ; 0.047) | 0.007 | 0.0062 |
| miR-450a-5p | -0.376 | (-0.716 ; -0.036) | -0.292 | 0.0070 |
| miR-604 | 0.020 | (-0.072 ; 0.113) | 0.026 | 0.0086 |
| miR-205-5p | -6.677 | (-14.421 ; 1.068) | -1.164 | 0.0192 |
| miR-551a | -0.677 | (-1.092 ; -0.261) | -0.636 | 0.0228 |
| miR-203a | -2.526 | (-6.063 ; 1.011) | -0221 | 0.0325 |
| miR-548l | -0.045 | (-0.111 ; 0.020) | 0.027 | 0.0354 |
| miR-424-5p | -2.292 | (-9.777 ; 5.193) | -3.939 | 0.0361 |
| miR-627-5p | -0.265 | (-0.450 ; -0.080) | 0.004 | 0.0392 |
| miR-629-3p | -0.241 | (-0.412 ; -0.070) | -0.288 | 0.0412 |
| miR-141-3p | -1.590 | (-2.882 ; -0.297) | -0.952 | 0.0439 |

Supplementary Table S3 MicroRNAs with significantly different levels in umbilical cord blood of mothers with pregnancy weight gain lesser than 12 kg.

| MiRNA | FC | 95% Confidence of FC | Median difference | p-value |
| --- | --- | --- | --- | --- |
| miR−138−5p | 0.356 | (-0.003 ; 0.715) | 0.317 | 0.0387 |
| miR−760 | 0.282 | (0.002 ; 0.563) | 0.223 | 0.0170 |
| miR−9−3p | 0.762 | (-0.250 ; 1.773) | 0.299 | 0.0144 |
| miR−548c−5p | 0.435 | (-0.139 ; 1.008) | 0.220 | 0.0449 |
| miR−1260a | 1.985 | (0.006 ; 3.965) | 0.673 | 0.0144 |
| miR−145−3p | 0.582 | (0.035 ; 1.129) | 0.415 | 0.0387 |
| miR−1224−3p | -0.023 | (-0.053 ; 0.007) | -0.013 | 0.0135 |
| miR−34a−3p | 0.578 | (-0.438 ; 1.594) | 0.519 | 0.0172 |
| miR−320d | 6.492 | (0.378 ; 12.605) | 1.978 | 0.0332 |

Supplementary Table S4 MicroRNAs with significantly different levels in umbilical cord blood associated with maternal blood type according to other blood types.

| MiRNA | FC | 95% Confidence of FC | Median difference | p-value |
| --- | --- | --- | --- | --- |
| Blood type A: |  |  |  |  |
| miR−380−5p | -0.199 | (-0.334 ; -0.063) | -0.207 | 0.0279 |
| miR−92a−1−5p | -0.182 | (-0.308 ; -0.055) | -0.174 | 0.0244 |
| Blood type B: |  |  |  |  |
| miR−760 | 0.461 | (-0.618 ; 1.540) | 0.258 | 0.0311 |
| miR−10b−5p | 15.610 | (-12.655 ; 43.875) | 15.412 | 0.0144 |
| miR−34b−3p | 0.116 | (-0.182 ; 0.415) | 0.037 | 0.0152 |
| miR−145−5p | 38.409 | (-39.009 ; 115.827) | 34.348 | 0.0247 |
| miR−153−3p | 0.123 | (-0.025 ; 0.271) | 0.131 | 0.0068 |
| miR−548c−5p | 0.831 | (-1.556 ; 3.217) | 0.189 | 0.0071 |
| miR−511−5p | 0.370 | (-0.115 ; 0.855) | 0.450 | 0.0256 |
| miR−330−5p | 0.285 | (-0.372 ; 0.943) | 0.181 | 0.0422 |
| miR−24−1−5p | 0.231 | (-0.033 ; 0.496) | 0.232 | 0.0188 |
| let−7b−3p | 3.149 | (-1.461 ; 7.760) | 2.234 | 0.0344 |
| let−7f−2−3p | 0.534 | (-0.560 ; 1.628) | 0.304 | 0.0386 |
| Blood type AB: |  |  |  |  |
| miR−595 | 0.011 | (-0.002 ; 0.023) | 0.011 | 0.0037 |
| miR−431−3p | 0.102 | (-0.019 ; 0.223) | 0.207 | 0.0437 |
| Blood type 0: |  |  |  |  |
| miR−641 | 0.074 | (-0.036 ; 0.184) | 0.076 | 0.0245 |
| miR−548h−5p | 0.026 | (-0.030 ; 0.083) | 0.006 | 0.0060 |

Supplementary Table S5 MicroRNAs with significantly different levels in umbilical cord blood of Rh-positive mothers.

| MiRNA | FC | 95% Confidence of FC | Median difference | p-value |
| --- | --- | --- | --- | --- |
| miR−141−3p | 1.617 | (0.235 ; 2.999) | 0.867 | 0.0471 |
| miR−188−5p | 0.216 | (0.104 ; 0.329) | 0.168 | 0.0075 |
| miR−211−5p | 0.059 | (0.010 ; 0.107) | 0.019 | 0.0388 |
| miR−205−5p | 6.726 | (-1.512 ; 14.963) | 1.403 | 0.0330 |
| miR−150−5p | 111.483 | (31.886 ; 191.08) | 111.499 | 0.0330 |
| miR−181c−5p | 0.131 | (-0.009 ; 0.270) | 0.076 | 0.0233 |
| miR−124−3p | 0.064 | (0.009 ; 0.120) | 0.025 | 0.0186 |
| miR−514a−3p | -0.024 | (-0.066 ; 0.018) | -0.002 | 0.0435 |
| miR−449b−5p | -0.003 | (-0.014 ; 0.008) | -0.003 | 0.0239 |
| miR−142−5p | 4.434 | (0.385 ; 8.483) | 3.076 | 0.0471 |
| miR−15b−5p | 12.884 | (4.252 ; 21.516) | 9.082 | 0.0471 |
| miR−641 | -0.056 | (-0.147 ; 0.034) | -0.042 | 0.0383 |
| miR−1269a | 0.021 | (0.006 ; 0.036) | 0.013 | 0.0281 |
| miR−1260a | 1.556 | (0.148 ; 2.964) | 0.767 | 0.0273 |
| miR−548l | -0.044 | (-0.134 ; 0.047) | -0.076 | 0.0140 |
| miR−518d−3p | 0.010 | (0.004 ; 0.016) | 0.002 | 0.0422 |
| miR−27a−5p | 0.265 | (0.033 ; 0.496) | 0.308 | 0.0395 |

Supplementary Table S6 MicroRNAs with significantly different levels in umbilical cord blood of mothers with allergies.

| MiRNA | FC | 95% Confidence of FC | Median difference | p-value |
| --- | --- | --- | --- | --- |
| miR−181d−5p | 0.259 | (-0.054 ; 0.573) | 0.163 | 0.0332 |
| miR−545−3p | 0.118 | (0.049 ; 0.187) | 0.119 | 0.0029 |
| miR−371a−3p | -0.061 | (-0.184 ; 0.062) | -0.012 | 0.0480 |
| miR−96−5p | -0.021 | (-0.115 ; 0.073) | -0.065 | 0.0454 |
| miR−216a−5p | -0.043 | (-0.118 ; 0.032) | -0.002 | 0.0405 |
| miR−153−3p | 0.062 | (0.012 ; 0.113) | 0.041 | 0.0148 |
| miR−632 | 0.070 | (-0.030 ; 0.170) | 0.036 | 0.0359 |

Supplementary Table S7 MicroRNAs with significantly different levels in umbilical cord blood of mothers with addictive substance abuse.

| MiRNA | FC | 95% Confidence of FC | Median difference | p-value |
| --- | --- | --- | --- | --- |
| miR−760 | -0.180 | (-0.424 ; 0.065) | -0.103 | 0.0441 |
| miR−377−3p | -0.137 | (-0.271 ; -0.002) | -0.109 | 0.0408 |
| miR−138−1−3p | 0.096 | (-0.009 ; 0.201) | 0.158 | 0.0239 |
| miR−33b−3p | 0.066 | (-0.021 ; 0.153) | 0.007 | 0.0376 |

Supplementary Table S8 MicroRNAs with significantly different levels in umbilical cord blood of mothers who did smoke during pregnancy (a) and stop smoking within pregnancy (b).

| a) |  |  |  |  |
| --- | --- | --- | --- | --- |
| MiRNA | FC | 95% Confidence of FC | Median difference | p-value |
| miR−129−5p | 0.032 | (-0.040 ; 0.105) | 0.047 | 0.0216 |
| miR−30b−3p | 0.011 | (-0.017 ; 0.039) | 0.013 | 0.0352 |
| miR−187−3p | 0.247 | (-0.492 ; 0.985) | 0.271 | 0.0383 |
| miR−507 | 0.111 | (-0.335 ; 0.557) | 0.016 | 0.0232 |
| miR−520b | 0.030 | (-0.034 ; 0.093) | 0.042 | 0.0006 |
| miR−33b−3p | 0.123 | (-0.215 ; 0.461) | 0.115 | 0.0146 |
|  |  |  |  |  |
| b) |  |  |  |  |
| MiRNA | FC | 95% Confidence of FC | Median difference | p-value |
| miR-138-1-3p | 0.146 | (0.020 ; 0.273) | 0.049 | 0.0156 |
| miR-760 | -0.241 | (-0.462 ; -0.021) | -0.079 | 0.0347 |
